# Genes Involved in Biofilm Matrix Formation of the Food Spoiler *Pseudomonas fluorescens* PF07

**DOI:** 10.3389/fmicb.2022.881043

**Published:** 2022-06-06

**Authors:** Miao Guo, Siqi Tan, Junli Zhu, Aihua Sun, Peng Du, Xiaoxiang Liu

**Affiliations:** ^1^School of Basic Medical Sciences and Forensic Medicine, Hangzhou Medical College, Hangzhou, China; ^2^School of Public Health, Hangzhou Medical College, Hangzhou, China; ^3^College of Food Science and Biotechnology, Zhejiang Gongshang University, Hangzhou, China

**Keywords:** *Pseudomonas fluorescens*, biofilm, extracellular matrix, food spoiler, functional amyloid, RpoN, BrfA

## Abstract

The extracellular matrix is essential for the biofilm formation of food spoilers. *Pseudomonas fluorescens* PF07 is a previous isolate from spoiled marine fish; however, the genes involved in the extracellular matrix formation of PF07 biofilms remain poorly defined. In this study, PF07 formed a wrinkled macrocolony biofilm through the high production of extracellular matrix. The genes involved in biofilm matrix formation and regulation were screened and identified by RNA-seq-dependent transcriptomic analysis and gene knock-out analysis. The macrocolony biofilms of PF07 grown for 5 days (PF07_5d) were compared with those grown for 1 day (PF07_1d). A total of 1,403 genes were significantly differentially expressed during biofilm formation. These mainly include the genes related to biofilm matrix proteins, polysaccharides, rhamnolipids, secretion system, biofilm regulation, and metabolism. Among them, functional amyloid genes *fapABCDE* were highly upregulated in the mature biofilm, and the operon *fapA-E* had a –24/–12 promoter dependent on the sigma factor RpoN. Moreover, the RNA-seq analyses of the *rpoN* mutant, compared with PF07, revealed 159 genes were differentially expressed in the macrocolony biofilms, and *fapA-E* genes were positively regulated by RpoN. In addition, the deletion mutants of *fapC*, *rpoN*, and *brfA* (a novel gene coding for an RpoN-dependent transcriptional regulator) were defective in forming mature macrocolony biofilms, solid surface-associated (SSA) biofilms, and pellicles, and they showed significantly reduced biofilm matrices. The *fap* genes were significantly downregulated in Δ*brfA*, as in Δ*rpoN*. These findings suggest that the functional amyloid Fap is the main component of PF07 biofilm matrices, and RpoN may directly regulate the transcription of *fap* genes, in conjunction with BrfA. These genes may serve as potential molecular targets for screening new anti-biofilm agents or for biofilm detection in food environments.

## Introduction

Biofilms are complex communities embedded in a self-produced extracellular matrix composed of proteins, exopolysaccharides, and extracellular DNAs (Flemming and Wingender, 2010). Bacteria form different types of biofilms depending on the environment. They include macrocolony biofilms that form on semi-solid agar plates for extended time, pellicles that form at the air–liquid interface of a standing culture, and solid surface-associated (SSA) submerged biofilms that formed on abiotic solid surfaces (Serra and Hengge, 2014). During food production, processing, and distribution, bacteria can form macrocolony biofilms or pellicles on the surfaces of various food materials and can form SSA biofilms on the surfaces of food industry equipment (Galié et al., 2018; Alvarez-Ordóñez et al., 2019). Biofilm formation provides physical resistance, mechanical resistance, or chemical protection to the bacterial cells, creating a serious challenge for the food industry in removing these food spoilers and pathogens (Bridier et al., 2015).

The extracellular matrix is essential for the biofilm formation of bacteria (Flemming and Wingender, 2010). The composition of the extracellular matrix formed by different strains varies greatly due to the genetic background and environment conditions. In the model bacterium *Escherichia coli* K12, amyloid curli fibers and cellulose are main extracellular matrix components (Mika and Hengge, 2014). The opportunistic pathogen *Pseudomonas aeruginosa* strains PAO1 and PA14 use polysaccharides Psl or Pel as the main components of the extracellular matrices, respectively (Ma et al., 2009; Jennings et al., 2015). The rhizobacteria *P*. *fluorescens* strains Pf0-1 and SBW25 produce adhesion protein LapA and cellulose Wss as the key components of the extracellular matrices, respectively (Spiers et al., 2003; Fazli et al., 2014). In *P*. *fluorescens* UK4 isolated from a reservoir, the functional amyloid Fap was identified from SSA biofilms (Dueholm et al., 2010). Recombinant overexpression of *fap* genes resulted in highly aggregative phenotypes with enhanced biofilm forming capacity in *Pseudomonas* strains; however, the Δ*fap* mutants formed SSA biofilms similar to the wild types, indicating that Fap is not an absolute requirement for biofilm formation of these wild-type strains under the experimental conditions (Dueholm et al., 2013; Zeng et al., 2015). Several other extracellular matrix components of *Pseudomonas* have been described using *in silico* methods, such as Flp/Tad pilus and polysaccharide poly-*N*-acetyl-glucosamine (PNAG) (Blanco-Romero et al., 2020).

Biofilm matrix production is controlled by complex regulatory networks that integrate multiple environmental signals by transcription factors, the second messenger cyclic diguanylate monophosphate (c-di-GMP), and sRNA (Mika and Hengge, 2014). Transcription factors are key regulators for the transcription of biofilm matrix genes. In *E*. *coli* K12, the transcription factor CsgD directly regulates the transcription of *csgBAC* coding for Curli (Mika and Hengge, 2014). In *P*. *aeruginosa* PA14, the transcription factor FleQ directly regulates the transcription of the *pel* genes (Hickman and Harwood, 2008). In *P*. *fluorescens* Pf0-1 and SBW25, the direct transcription factors for *lapA* and *wss* are currently unknown. In *P*. *fluorescens* UK4 cultured in liquid fish juice at 4^°^C, the transcription of *fap* genes were positively regulated by the alternative sigma factor RpoN, but the understanding of the transcription regulation of the *fap* gene in biofilm remains limited (Liu et al., 2021). In addition, c-di-GMP has effects on the production of biofilm matrices. C-di-GMP is produced and degraded by multiple diguanylate cyclases (DGCs) and phosphodiesterases (PDEs), respectively. The production of curli amyloids in *E*. *coli* biofilms, Pel polysaccharides in *P*. *aeruginosa* biofilms, and LapA, and Wss in *P*. *fluorescens* biofilms are all regulated by c-di-GMP (Serra et al., 2013; Okegbe et al., 2017; Collins et al., 2020). Moreover, sRNA is also involved in the regulation of the biofilm matrix production (Fazli et al., 2014; Mika and Hengge, 2014).

*Pseudomonas* spp. contribute significantly to the spoilage of food. They are the predominant spoilers of proteinaceous raw foods stored under aerobic refrigerated conditions (Remenant et al., 2015). *P*. *fluorescens* is currently recognized as one of the most abundant spoilers of this genus, and it has been extensively isolated from dairy, fish, and meat (Nogarol et al., 2013; Wang et al., 2018b,^2020^). *P*. *fluorescens* is a strong biofilm producer, and the extracellular matrix of the biofilm has a structural role, enhancing the persistence of these biofilms in the food industry (Galié et al., 2018; Zhu et al., 2019). Particularly in the fish industries, *P*. *fluorescens* adheres to the surface of processing equipment in the form of a biofilm, even after cleaning and disinfecting the equipment (Bagge-Ravn et al., 2003; Langsrud et al., 2016). Moreover, the extracellular matrix of its biofilm can provide shelter for foodborne pathogen *Listeria monocytogenes* (Puga et al., 2018).

The *P*. *fluorescens* PF07 strain was isolated from spoiled large yellow croaker in our previous work, and it exhibited high spoilage potential (Wang et al., 2021). However, the genetic basis for the formation of extracellular matrices in PF07 biofilms remains poorly defined. In this study, PF07 was identified as a strong biofilm producer, as it can form dense macrocolony biofilms, SSA biofilms, and pellicles. The genes involved in biofilm matrix formation and regulation were screened and identified. First, RNA-seq-dependent transcriptomic analysis of macrocolony biofilms (PF07_5d vs. PF07_1d, Δ*rpoN* vs. PF07) was performed to identify genes related to the biofilm matrix formation. Moreover, by promoter, gene knock-out, and biofilm phenotype analyses, functional amyloid Fap is the main component of PF07 biofilm matrices, and RpoN may directly regulate the transcription of *fap* genes, in conjunction with a novel RpoN-dependent transcriptional regulator D7M10_RS14495, which we have named BrfA for “biofilm regulator of *fap*.”

## Materials and Methods

### Strains and Growth Conditions

All bacterial strains and plasmids used in this study are presented in Table 1. *P*. *fluorescens* PF07 and its mutants were grown in Luria-Bertani (LB) or tryptone broth (10 g/L tryptone) medium at 28°C.

**TABLE 1:** Strains and plasmids.

Strains or plasmids	Characteristics	References or source
*Escherichia coli*		
β2163	F^–^, RP4-2-Tc:Mu Δ*dapA*:(erm-pir), Km^r^Em^r^	Demarre et al., 2005
*Pseudomonas fluorescens*		
PF07	An isolate from spoiled large yellow croaker	Wang et al., 2021
Δ*rpoN*	PF07, *rpoN* deletion mutant	This study
Δ*fapC*	PF07, *fapC* deletion mutant	This study
Δ*brfA*	PF07, *brfA* deletion mutant	This study
Plasmids		
pLP12Cm	*oriT*_*RP*4_ *oriV*_*R*6K_ *vmi*480 P_*BAD*_, Cm^r^	Laboratory collection
pLP12Cm-*rpoN*	pLP12Cm derivative containing *rpoN* ORF bp 1-215 fused to bp 1474-1494, Cm^r^	This study
pLP12Cm-*fapC*	pLP12Cm derivative containing *fapC* ORF bp 1-3 fused to bp 680-756, Cm^r^	This study
pLP12Cm-*brfA*	pLP12Cm derivative containing *brfA* ORF bp 1-81 fused to bp 1216-1326, Cm^r^	This study

### Mutant Construction

The deletion mutants Δ*rpoN*, Δ*fapC*, and Δ*brfA* of *P*. *fluorescens* PF07 were constructed by double-crossover allelic exchange, according to the method by Liu et al. (2018). All primers used in the mutant construction are listed in Supplementary Table 1. Briefly, two fragments flanking *rpoN* were amplified by PCR with 07*rpoN*-MF1/MR1 and 07*rpoN*-MF2/MR2 primers, respectively. The sequences of the two primers 07*rpoN*-MR1 and 07*rpoN*-MF2 were reverse and complementary. The two fragments were purified and fused in subsequent PCR reactions using 07*rpoN*-MF1/MR2 primers. In the same way, two fragments flanking the *fapC* or *brfA* were amplified and fused using the corresponding primers of each gene. The fused segment of the *rpoN* gene with a deletion of 1,258 bp in its 1,494-bp open reading frame (ORF), the fused segment of the *fapC* gene with a deletion of 676 bp in its 756-bp ORF, and the fused segment of the *brfA* gene with a deletion of 1,134 bp in its 1,326-bp ORF, were cloned into the suicide vector pLP12Cm using a ClonExpress II One Step Cloning Kit (Vazyme, China), respectively. The recombinant plasmids pLP12Cm-*rpoN*, pLP12Cm-*fapC*, and pLP12Cm-*brfA* were transferred from *E*. *coli* β2163 (Demarre et al., 2005) into *P*. *fluorescens* PF07 by conjugation. The single crossover transconjugants were selected on LB agar medium containing 20 μg/mL chloramphenicol and 0.3% D-glucose at 28°C. L-arabinose can induce the expression of the virulence gene *vmi480* on the suicide vector; thus, the second crossover mutants were screened on LB agar with 0.4% L-arabinose. The PF07 Δ*rpoN*, Δ*fapC*, and Δ*brfA* mutants were confirmed *via* PCR, using external primer pairs *rpoN*-TF/TR, *fapC*-TF/TR, and *brfA*-TF/TR, respectively. The deletion mutants were further confirmed by sequencing.

### RNA-seq Analyses

The overnight cultures of PF07 and the *rpoN* mutant (5 μL each) were spotted on tryptone plates (1% tryptone, 1% agar). After 1 day of incubation at 28°C, 24 colonies of PF07 were gently scraped from six tryptone plates and mixed in 10 mL of 0.9% NaCl. The sample was harvested by centrifugation at 7,000 × *g* for 10 min and frozen in liquid nitrogen prior to RNA isolation. After 5 days of incubation, macrocolonies of PF07 and the *rpoN* mutant were collected using the same method, respectively. Two biological replicates were prepared for each treatment. The obtained samples were sent to Novogene Co., Ltd. (Beijing, China) for RNA-seq analysis. Total RNA was isolated from the frozen samples using an RNAprep Pure Cell/Bacteria Kit and a miRcute miRNA Isolation Kit (Tiangen, China). The concentration and quality of total RNA were validated using a NanoDrop spectrophotometer (Thermo, United States) and Agilent 2100 Bioanalyzer (Agilent, United States), and the qualified RNA samples were used as input materials. mRNA was purified from total RNA using probes to remove rRNA. Then, strand-specific RNA sequencing libraries were constructed using NEBNext Ultra Directional RNA Library Prep Kit for Illumina (NEB, United States) according to the manufacturer’s introductions. Following validation with an Agilent Bioanalyzer 2100 system (Agilent, United States), the libraries were sequenced on an Illumina Hiseq platform and paired-end reads were generated. The raw reads were filtered to remove adapter-containing reads, high N rate reads (N rates > 10%), and low-quality reads (50% bases with Q-score ≤ 5) using in-house Perl scripts. The genome sequence of *P*. *fluorescens* PF08, an isolate from refrigerated turbot, was used as the reference genome,^1^ and the obtained clean reads were mapped to the reference genome using Bowtie2 v2.2.3 (Langmead and Salzberg, 2012). The novel gene and sRNA were identified by Rockhopper (McClure et al., 2013). The gene expression levels were quantified, and differential expression analysis was performed using DESeq2 (Love et al., 2014). Genes with an adjusted *P*-value < 0.05 and | log_2_ fold change| ≥ 1 were considered to be differentially expressed (Wang et al., 2018a; Liu et al., 2019).

### Quantitative Reverse Transcription-PCR Assays

qRT-PCR analyses were performed with RNA samples under the same culture conditions used for the RNA-seq experiments. Total RNA was isolated with a TRIzol Plus RNA Purification Kit (Invitrogen, United States) and treated with RNase-Free DNase Set (Qiagen, Germany) to remove the contaminating DNA. cDNA was synthesized using the SuperScript™ III First-Strand Synthesis SuperMix (Invitrogen, United States), and qRT-PCR analyses were conducted using a CFX384 Touch Real-Time PCR Detection System (Bio-Rad, United States) with the Power SYBR1 Green PCR Master Mix (Applied Biosystems, United States). Primers for qRT-PCR are listed in Supplementary Table 2. The 16S rRNA gene was used as a reference control for sample normalization. The relative expression of the target genes was calculated using the 2^–ΔΔ*Ct*^ method (Livak and Schmittgen, 2001). Three biological replicates were prepared, and samples were run in triplicate.

### Rapid Amplification of 5′-cDNA Ends

To identify the 5′-end of a gene, rapid amplification of 5′-cDNA ends (5′-RACE) was performed according to a method described previously (Liu et al., 2018). Briefly, the Vaccinia Capping System (NEB, United States) and GeneRacer Kit (Invitrogen, United States) were used according to the manufacturers’ instructions. The 5′-ends of the transcripts were capped with the Vaccinia Capping System, and the GeneRacer Kit ensured the amplification of only full-length transcripts *via* the elimination of truncated messages from the amplification process. The *fapA* specific primers used for 5′-RACE are listed in Supplementary Table 1. The 5′ RACE products were directly sequenced to identify the 5′-end of *fapA*.

### Observation of Macrocolony Biofilm

Macrocolony biofilms were observed by Congo red assay and transmission electron microscopy (TEM), using a previously reported method (Liu et al., 2019). Congo red plates (1% tryptone, 1% agar, 40 μg/mL Congo red, and 10 μg/mL Coomassie brilliant blue G250) were used to assess macrocolony biofilm color and morphology. Cells of PF07 and the mutants were grown overnight in liquid LB medium with shaking at 28°C. A total of 5 μL of the overnight cultures was spotted on Congo red plates. The plates were incubated at 28°C for 1, 3, 5, and 7 days to observe the macrocolony morphology. For TEM analysis, the macrocolonies on day 5 were gently scraped from a tryptone plate (1% tryptone, 1% agar) and fixed in 2.5% glutaraldehyde for more than 4 h at room temperature. Then, the macrocolony biofilms were postfixed, dehydrated, embedded in TAAB resin (TAAB Laboratories, United Kingdom), ultrathin-sectioned, stained, and observed *via* TEM (Hitachi H-600, Japan).

### Pellicle Formation Assay

Overnight cultures of PF07 and the mutants (6 μL each) were added into 6 mL of fresh sterile tryptone broth. The dilution culture was statically grown in a glass tube at 28°C for 3 days. Pellicles were assayed by visual inspection of the air–liquid interface of the standing culture.

### Crystal Violet Assay for Microplate Biofilm

Quantification of biofilms grown in microplates was performed as described by Liu et al. (2021). Tryptone broth was used as the growth medium, and cultures were incubated at 28°C for 6, 12, 24, and 48 h with shaking (150 rpm). After crystal violet staining, the absorbance at 595 nm was measured using a microplate reader (Infinite 200, Tecan, Switzerland). Eight individual samples were assessed for each strain at each time point, and the experiment was repeated at least twice.

### Confocal Laser Scanning Microscopy Observation of Biofilms on Glass Slips

Overnight cultures were inoculated into 12-well dishes containing tryptone broth at a 1:1,000 ratio. Glass coverslips (20 mm × 20 mm) were partially submerged in the medium and grown at 28°C with shaking (150 rpm). After incubation for 24 h, the glass slips were washed two times with 0.9% NaCl. The biofilm on the glass slips was successively stained with 150 ng/mL 4′,6-diamidino-2-phenylindole (DAPI) for 20 min and 2 mg/mL Congo red for 20 min in the dark at room temperature (Larsen et al., 2007). The coverslips were washed and dried, and then a drop of liquid Antifade mounting medium (Beyotime, China) was placed on the coverslips to prevent fluorescence quenching. All the coverslips with stained biofilms were placed on slides and imaged with a Nikon Eclipse Ti2 confocal laser scanning microscopy (CLSM) (Nikon, Japan) and a 63 × oil objective. Fluorescence signals were recorded in the blue channel (excitation 405 nm, emission 425–475 nm) for DAPI and in the red channel (excitation 561 nm, emission 570–620 nm) for Congo red, where at least 10 fields of view were captured. NIS-Elements AR confocal software was used to analyze the biofilm images, allowing for collection of z-stacks.

### Growth Determination

Overnight cultures were diluted 1:100 into 150 mL fresh tryptone broth medium, and cultivated at 28°C with constant shaking (220 rpm) for 24 h. The optical absorbance at 600 nm was measured at 4-h intervals. Three independent experiments were performed, and the mean optical absorbance values at each time point were used to determine the growth rate.

## Results

### Overview of Gene Expression Profiles During Macrocolony Biofilm Formation

**FIGURE 1:**
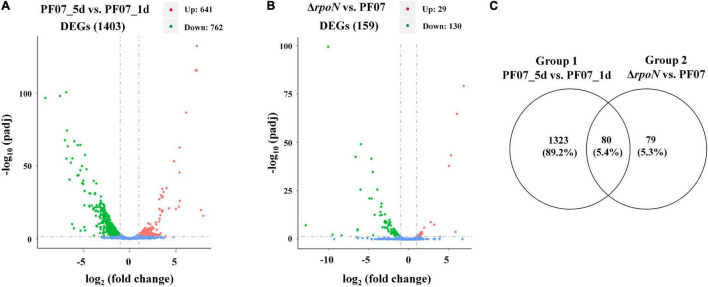
DEGs from RNA-seq analyses. **(A)** DEGs between macrocolony biofilms of PF07_5d and PF07_1d (Group 1). **(B)** DEGs between macrocolony biofilms of Δ*rpoN* and PF07 after 5 days of incubation (Group 2). The horizontal axis represents fold changes of gene expression, and the vertical axis represents the level of statistical significance. The red dots represent significantly upregulated genes, and the green dots represent significantly downregulated genes. **(C)** Venn diagram of Group 1 and Group 2. This figure was drawn using Venny 2.0. (http://bioinfogp.cnb.csic.es/tools/venny/index.html).

*P*. *fluorescens* PF07 is a potent biofilm producer, and it formed a red, wrinkled macrocolony on a Congo red plate after at least 3 days of growth. The red and wrinkled macrocolony of bacteria often results from the production of large amounts of extracellular matrices (Friedman and Kolter, 2004). In the present work, RNA-seq-dependent transcriptomic analysis was utilized to identify genes involved in biofilm matrix formation and regulation. The macrocolony biofilms of PF07 grown on tryptone plates for 5 days (PF07_5d) were compared with those grown for 1 day (PF07_1d). RpoN is an important sigma factor involved in biofilm regulation of bacteria (Xu et al., 2019; Liu et al., 2021); therefore, the *rpoN* mutant of PF07 was constructed to determine the regulatory role of RpoN in biofilm matrix formation (Supplementary Figure 1). The raw sequencing data of RNA-seq were deposited in the Sequence Read Archive,^2^ with an accession number PRJNA800221. After the raw reads were filtered, the clean reads were mapped to the reference genome sequence of *P*. *fluorescens* PF08, with a total mapped rate of at least 96.31% for all samples. The high mapped rates suggest that PF07 strain is very closely related to the reference strain PF08, an isolate from refrigerated turbot (Xu et al., 2021). The obtained read data and mapping data are shown in Supplementary Tables 3, 4. According to the screening criteria for differentially expressed genes (DEGs) (| log2 fold change| ≥ 1, padj ≤ 0.05), a total of 1,403 DEGs were identified, including 641 significantly upregulated genes and 762 significantly downregulated genes in PF07_5d, compared with PF07_1d (Figure 1A). In addition, 159 DEGs were identified by comparing the macrocolony biofilms of *rpoN* mutant and PF07 on day 5, including 29 significantly upregulated genes and 130 significantly downregulated genes (Figure 1B). The detailed information on the two groups of DEGs is summarized in Supplementary Tables 5, 6. The relationship between the two groups of DEGs was further analyzed, and a total of 80 DEGs were obtained in the overlap of the two groups (Figure 1C).

### Functional Analysis of Differentially Expressed Genes

Based on the functional annotation, chromosomal location, and expression pattern, the representative DEG clusters or DEGs are listed in Table 2, and the results were validated by qRT-PCR. The qRT-PCR results agreed with the expression patterns from the RNA-seq analyses, except for *pgaABCD*, a gene cluster related to the biosynthesis of the extracellular polysaccharide PNAG. The main components of the biofilm matrix are proteins or extracellular polysaccharides, and the genes coding for these components are usually present in the form of gene clusters (Blanco-Romero et al., 2020). The proteinaceous components of the biofilm matrix mainly include secreted extracellular proteins, cell surface adhesins and protein subunits of cell appendages, such as flagella and pili (Fong and Yildiz, 2015). As shown in Table 2, the main DEGs related to matrix proteins are *fapABCDE*, *flp/tad*, and flagellar genes. Interestingly, the gene cluster *fapA-E*, encoding functional amyloids, was significantly upregulated in PF07_5d compared with PF07_1d, and significantly downregulated in Δ*rpoN* compared with PF07 (Table 2). This gene cluster is likely to be involved in the formation of biofilm matrix and is positively regulated by RpoN. However, the *flp/tad* gene cluster, encoding a special type IVb pilus, was significantly downregulated in PF07_5d compared with PF07_1d. The expression of genes associated with flagella was also significantly downregulated in PF07_5d, compared with PF07_1d, as well as in Δ*rpoN* compared with PF07 (Table 2). In addition to the matrix protein genes, the representative DEGs related to polysaccharide metabolism are *pgaABCD* and glycogen metabolism genes. According to the results of RNA-seq, the *pga* gene cluster was upregulated in PF07_5d compared with PF07_1d, but the qRT-PCR results showed downregulation. The discrepancy suggests that the expression of *pga* genes in biofilms may lack repeatability as the qRT-PCR tests were performed using independently extracted RNAs. The expression of the genes associated with glycogen metabolism was also downregulated in PF07_5d compared with PF07_1d. Moreover, the gene cluster *rhlABRI* was significantly downregulated in PF07_5d compared with PF07_1d, but upregulated in the *rpoN* mutant compared with PF07_1d. The gene cluster encodes enzymes (RhlAB) for rhamnolipid synthesis and the quorum sensing system (RhlRI). Finally, the bacterial secretion system is also likely to be associated with biofilm formation. As shown in Table 2, the type II secretion system was upregulated, while another possible secretion system was downregulated at the transcription level in PF07_5d, compared with PF07_1d.

**TABLE 2:** Representative DEGs from RNA-seq analysis and qRT-PCR verification.

			PF07_5d vs. PF07_1d^c^		Δ*rpoN* vs. PF07^d^	
			RNA-seq	qRT-PCR	RNA-seq^e^	qRT-PCR^f^
			
Gene cluster or gene^a^	N^b^	Function description	log_2_ (fold change) (adjusted *P*-value)	log_2_ (fold change) (*P*-value)	log_2_ (fold change) (adjusted *P*-value)	log_2_ (fold change) (*P*-value)
**Matrix Proteins**						
D7M10_RS14465-**75**-85 (*fapABCDE*)	5	Functional amyloid Fap biosynthesis	3.4 ∼ 5.4 (<1.1E-30)	2.3 ± 0.2 (2.1E-13)	–3.6 ∼ –10.1 (<3.0E-13)	–5.3 ± 0.5 (8.7E-16)
D7M10_RS03055–20, D7M10_**RS03060**–70	11	Flp/Tad pilus formation	–1.7 ∼ –3.9 (<0.01)	–2.6 ± 0.2 (8.1E-17)	–	ND
D7M10_RS08115–**35** (*fliCflaGfliDST*)	5	Flagellar biosynthesis	–1.3 ∼ –2.7 (<9.6E-04)	–1.3 ± 0.6 (4.8E-06)	–1.7 ∼ –4.9 (<0.03)	–1.2 ± 0.5 (6.8E-06)
D7M10_RS20375–**65** (*flgCDE*)	3	Flagellar biosynthesis	–1.2 ∼ –1.4 (<5.1E-03)	–0.5 ± 0.05 (2.1E-12)	–2.7 ∼ —3.5 (<1.2E-06)	–3.6 ± 0.5 (2.8E-13)
**Polysaccharides**						
D7M10_**RS00760**–75 (*pgaABCD*)	4	PNAG polysaccharide biosynthesis	2.6 ∼ 3.5 (<6.4E-08)	–1.0 ± 0.3 (5.2E-09)	–	ND
D7M10_RS14080-**105**, D7M10_RS14155-50	8	Glycogen metabolism	–1.1 ∼ –2.9 (<2.9E-04)	–0.5 ± 0.2 (5.1E-06)	–	ND
**Rhamnolipid**						
D7M10_RS11580-**65** (*rhlABRI*)	4	Rhamnolipid synthesis and quorum sensing system	–5.1 ∼ –7.5 (<3.8E-53)	–6.0 ± 0.4 (5.5E-17)	5.1 ∼ 6.9 (<1.5E-38)	1.0 ± 0.1 (2.8E-04)
**Secretion systems**						
D7M10_**RS10920**-45 (*gspKLMDEF)*	6	Type II secretion system	1.6 ∼ 2.8 (< 0.02)	2.5 ± 0.6 (8.2E-10)	–	ND
D7M10_RS03305-**15**-25	5	Secretion system	–2.3 ∼ –3.2 (<1.5E-14)	–1.2 ± 0.5 (8.5E-06)	–	ND
**Biofilm regulation**						
D7M10_RS14495	1	RpoN-dependent Fis family transcriptional regulator	2.0 (3.1E-11)	0.6 ± 0.3 (6.7E-04)	–	ND
D7M10_RS04965	1	Diguanylate cyclase	1.1 (2.0E-03)	1.2 ± 0.1 (2.5E-13)	–	ND
D7M10_RS06580	1	Diguanylate cyclase/ phosphodiesterase	–2.1 (3.6E-11)	–0.9 ± 0.3 (2.4E-06)	–	ND
D7M10_RS22095	1	Diguanylate cyclase/ phosphodiesterase	–2.2 (1.8E-13)	–1.0 ± 0.4 (2.3E-04)	–	ND
**Metabolism**						
D7M10_**RS00150**-65	4	Galactonate degradation	3.2 ∼ 4.0 (<6.5E-28)	1.4 ± 0.2 (3.3E-10)	–1.2 ∼ –1.8 (<0.03)	ND
D7M10_**RS13750**-85	8	Biosynthesis and secretion of extracellular protease	–1.4 ∼ –5.3 (<4.4E-04)	–7.5 ± 0.2 (4.1E-23)	–	ND
D7M10_RS21085-**100** (*arcDABC*)	4	Arginine degradation	–2.4 ∼ –2.7 (<4.6E-12)	–2.7 ± 0.3 (2.6E-14)	–	ND
**Function unknown**						
D7M10_**RS18830**-15	4	Function unknown	6.1 ∼ 7.2 (<1.9E-87)	4.1 ± 0.1 (6.4E-20)	–4.3 ∼ –6.4 (<0.01)	–1.9 ± 0.1 (5.6E-14)

*^a^The gene in a gene cluster used for qRT-PCR verification is highlighted in bold.*

*^b^N, number of genes.*

*^c^The macrocolony biofilms grown for 5 days (PF07_5d) were compared with those grown for 1 day (PF07_1d).*

*^d^The macrocolony biofilms of ΔrpoN were compared with PF07 after 5 days of incubation.*

*^e^The “-” symbol indicates that the expression of the gene was not detected or showed no significant difference.*

*^f^ND, not defined.*

Some genes that may be involved in the regulation of biofilm matrix synthesis were also identified (Table 2). A novel RpoN-dependent transcription factor D7M10_RS14495, which we named BrfA, was significantly upregulated in PF07_5d compared with PF07_1d. Some genes encoding diguanylate cyclase and phosphodiesterase (*D7M10_RS04965*, *D7M10_RS06580*, and *D7M10_RS22095)* were significantly altered during the formation of the biofilm. The second messenger c-di-GMP regulates the expression of biofilm matrix components in most bacteria, and the level of intracellular c-di-GMP concentration is controlled by diguanylate cyclase and phosphodiesterase (Fazli et al., 2014). In addition, the transcript levels of several novel sRNAs were also altered during the formation of biofilms (Supplementary Table 5). However, these novel sRNAs require further confirmation.

With regard to the usage of carbon and nitrogen sources, we found that genes related to galactose catabolism (*D7M10_RS00150-65*) were significantly upregulated in the mature biofilm, but genes related to amino acid utilization (*D7M10_RS13750-85*, *arcDABC*) were significantly downregulated during the biofilm formation. In addition, a gene cluster with unknown function (*D7M10_RS18830-15*) was highly upregulated in PF07_5d compared with PF07_1d, and significantly downregulated in Δ*rpoN* compared with PF07 (Table 2).

### Identification of RpoN-Dependent Promoter of *fapA*

**FIGURE 2:**
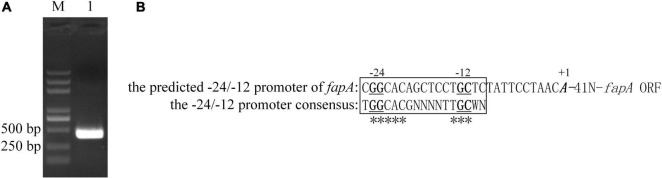
Transcription initiation site and RpoN-dependent promoter of *fapA*. **(A)** The 5′-RACE-PCR products of *fapA* (Lane 1). Lane M, DL2000 DNA marker (Takara, China). **(B)** The translation start site is indicated by bold and italic text. Nucleotides that were 100% conserved in all examined RpoN-dependent promoters by Barrios et al. (1999) are labeled with asterisks (*). The GG and GC elements are indicated by bold and underlined text.

The RNA-seq results indicated the gene cluster *fapA-E* was positively regulated by RpoN. RpoN recognizes promoter sequences with conserved GG and GC elements located –24 and –12 nucleotides upstream from the transcriptional start site (Barrios et al., 1999). According to chromosomal location and expression pattern, *fapA-E* are arrayed in an operon, and *fapA* is the first gene of the operon. The 5′-RACE method was performed to identify the transcriptional start site of *fapA*. As shown in Figure 2, a conserved –24/–12 element was present upstream from the transcriptional start site. This result suggests that *fapA-E* genes are directly regulated by RpoN.

### The Defects of Δ*fapC*, Δ*rpoN*, and Δ*brfA* in Forming Mature Macrocolony Biofilms

**FIGURE 3:**
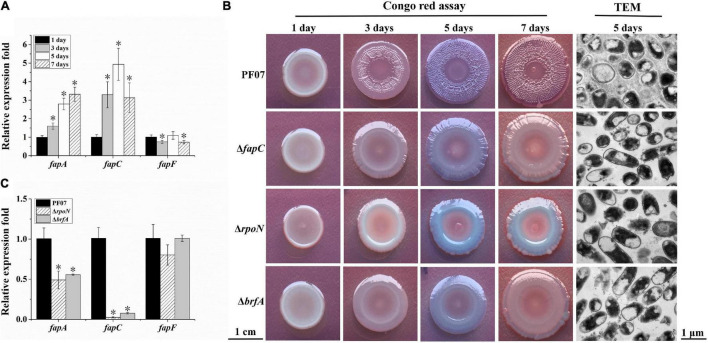
Expression analyses of *fap* genes and macrocolony biofilm observation. **(A)** Expression changes of *fap* genes over time for PF07 on tryptone plates. **(B)** Macrocolony biofilms observed by Congo red assay (scale bar = 1 cm) and TEM (scale bar = 1 μm). **(C)** Expression changes of *fap* genes in 5-day-old macrocolony biofilms of △*rpoN* and △*brfA*, compared to PF07. The expression levels of genes were assayed by qRT-PCR. Data are presented as the mean ± SD (*n* = 9). Significant differences compared to 1 day **(A)** or compared to PF07 **(C)** were analyzed using one-way ANOVA (analysis of variance), *P < 0.01.

The *fap* cluster contains six ORFs *fapABCDEF*. According to the results of the RNA-seq, the transcription of *fapA-E* was significantly upregulated during biofilm formation, but the expression of *fapF* was not upregulated (Table 2). The time-course analysis of *fap* gene expression during biofilm formation by qRT-PCR further supported the RNA-seq results (Figure 3A). These results suggest differences in the expression patterns of *fapF* and *fapA-E*. FapC is the main component of the Fap fibers (Bleem et al., 2018); thus, the *fapC* mutant was constructed to observe the role of the Fap amyloids during biofilm formation (Supplementary Figure 1). Biofilm morphology assays on Congo red plates were performed (Figure 3B). After 1 day of growth, the colonies of the *fapC* mutant were the same as the wild-type colonies and characterized as pale and smooth. After 3 days, the colonies of the wild type started to become red and wrinkled, but those of the *fapC* mutant remained pale and smooth. On days 5 and 7, the wild-type strain formed mature macrocolony biofilms, which were redder and more wrinkled than the *fapC* mutant. Additionally, we used TEM to visualize the structure of the macrocolony biofilms. After 5 days, the wild-type cells were encased in a basket-like matrix, whereas the mutant cells generated much less extracellular matrix (Figure 3B). These results suggest that PF07 can develop wrinkled macrocolony biofilms through the high production of the extracellular matrix, and that Fap amyloid is the main component of PF07 biofilm matrices.

Similar to the *fapC* mutant, the *rpoN* mutant also failed to form red and wrinkle macrocolonies, and it failed to generate an extracellular matrix in the biofilm (Figure 3B). The RNA-seq results show the cells in the macrocolony biofilm of the *rpoN* mutant significantly downregulated the transcription of *fapA-E* genes (Table 2). These results indicate that RpoN regulates the production of the biofilm matrix *via* regulation of the *fap* gene cluster.

Downstream of the *fapA-F* gene sequence of PF07, there is an adjacent reverse gene, *brfA* (*D7M10_RS14495*), which encodes an RpoN-dependent Fis family transcriptional regulator and is upregulated in mature biofilms (Table 2). The deletion mutant of *brfA* was constructed (Supplementary Figure 1), and Δ*brfA* showed macrocolony biofilms similar to Δ*fapC* and Δ*rpoN* (Figure 3B). In addition, the transcription of *fapA* and *fapC* was also significantly down-regulated in Δ*brfA* as in Δ*rpoN* (Figure 3C). These results suggest BrfA is likely to act in conjunction with RpoN to regulate the transcription of *fap* genes.

### The Defects of Δ*fapC*, Δ*rpoN*, and Δ*brfA* in Forming Pellicles and Solid Surface-Associated Biofilms

**FIGURE 4:**
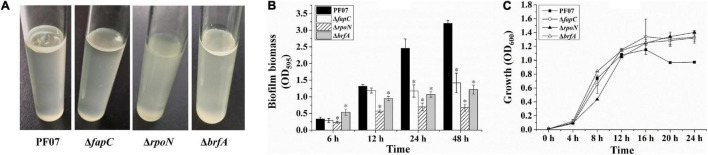
Pellicle and SSA biofilm formation of PF07, Δ*fapC*, Δ*rpoN*, and Δb*rfA*. **(A)** Pellicle formed at the air–liquid interface of the standing culture. **(B)** Crystal violet assay for SSA biofilms in microplates. The biofilm biomass was quantified by crystal violet staining. Data are expressed as mean ± SD of eight replicates. Significant differences between PF07 and the mutants were analyzed by one-way ANOVA, *P < 0.01. **(C)** The growth curves of PF07 and the mutants in tryptone broth medium with shaking. Data are expressed as mean ± SD of three replicates.

*P*. *fluorescens* PF07 formed robust pellicles when grown in standing culture (1% tryptone) after 3 days of growth. However, the mutants Δ*fapC*, Δ*rpoN*, and Δ*brfA* grew as turbid cultures and failed to form robust pellicles, especially the *rpoN* mutant (Figure 4A). Thus, the three genes are required for pellicle formation by *P*. *fluorescens* PF07.

The ability of the bacteria to form SSA biofilms in plastic microplates was analyzed by crystal violet staining. As shown in Figure 4B, after 6 h of growth, Δ*rpoN* showed a slight defect in the initial adhesion. After 12 h, the biofilm biomass of Δ*rpoN* and Δ*brfA* was significantly reduced compared with the wild type. After 24 and 48 h, the three mutants produced significantly less biofilm than the wild type, by at least 50%. To determine whether the three mutants had growth defects, their growth curves were plotted. As shown in Figure 4C, all the strains started to enter the stationary phase after 12 h of growth. During the logarithmic growth phase (4–12 h), the growth of Δ*rpoN* was slower than the other strains. After 16 h, the optical density of the wild type was lower than the mutants. These results suggest that *fapC*, *rpoN*, and *brfA* genes are required for SSA biofilm maturation in *P*. *fluorescens* PF07. However, FapC and BrfA may not be involved in the initial adhesion process of biofilm formation.

**FIGURE 5:**
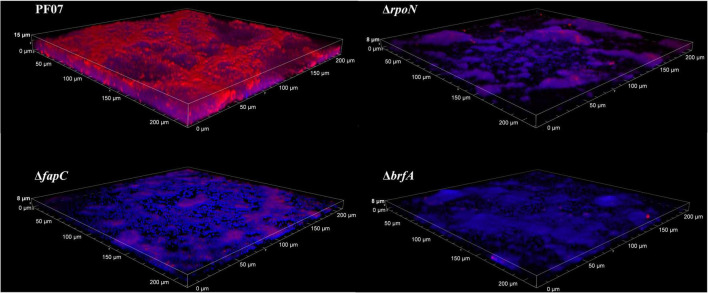
CLSM observation of biofilm on glass slips of PF07, Δ*fapC*, Δ*rpoN*, and Δb*rfA*. The biofilms were grown along the air-liquid interface of 20-mm glass coverslips. At 24 h the coverslips were removed, washed in 0.9% NaCl, and stained with DAPI for cells (blue) and Congo red for extracellular matrix (Red). Shown are representative images of each strain.

The biofilms formed on glass coverslips were stained with both Congo red and DAPI to show the structure of the biofilm by CLSM. Congo red is frequently used for amyloid and polysaccharide staining in biofilm matrices, and the DNA stain DAPI (blue) is used to visualize the bacterial cells (Larsen et al., 2007). As shown in Figure 5, PF07 formed thick and rough biofilms covering most of the surface after 24 h of incubation. The cells with blue fluorescence were wrapped and covered by extracellular matrices with red fluorescence. Compared to PF07, Δ*fapC*, Δ*rpoN*, and Δ*brfA* formed notably thinner biofilms that sparsely covered the surface, and the red fluorescent signal was significantly reduced. The CLSM results are consistent with the results of the crystal violet assay in plastic microplates. These results further confirm that Fap amyloid is the main component of the SSA biofilm matrices, and both RpoN and BrfA regulate the production of biofilm matrices by controlling the transcription of the *fap* genes.

## Discussion

The biofilm matrix encases the constituent cells, providing a scaffold for the biofilm architecture (Flemming and Wingender, 2010). In the present work, *P*. *fluorescens* PF07 developed wrinkled macrocolony biofilms on tryptone plates for an extended time through the high production of extracellular matrices. The genes differentially expressed during macrocolony biofilm formation may be related to extracellular matrix production. In addition, the *rpoN* mutant was deficient in forming wrinkled macrocolony biofilms and in producing extracellular matrices (Figure 3B). Thus, we explored the genes involved in biofilm matrix formation by RNA-seq-dependent transcriptomic analyses of macrocolony biofilm of *P*. *fluorescens* PF07 (PF07_5d vs. PF07_1d, Δ*rpoN* vs. PF07), and we identified several gene clusters or genes that may be associated with the formation and regulation of the biofilm matrix (Table 2).

The *fap* gene cluster is the most intriguing. Dueholm et al. (2010) first showed that the Fap amyloid in *Pseudomonas* is expressed from the gene cluster *fapABCDEF*. Detailed biophysical investigations of purified fibrils confirmed that FapC represents the major subunit of the mature Fap fibril, and FapB is a minor constituent. However, little is known about the expression regulation of *fap* genes. In the present work, the transcription of *fapA-E* genes was significantly induced during macrocolony biofilm formation of PF07, an isolate from spoiled fish (Table 2 and Figure 3A). In addition, we showed that the operon *fapABCDE* has an RpoN-dependent promoter, and RpoN may directly regulate the transcription of *fap* genes in PF07 (Figure 2). RpoN recognizes a characteristic –24/–12 promoter and requires an associated activator to initiate the transcription of specific genes (Bush and Dixon, 2012). The *brfA* gene is an adjacent reverse gene downstream of the *fapA-F* gene sequence of PF07, which encodes a novel RpoN-dependent Fis family transcription regulator and is upregulated in mature biofilms (Table 2). Moreover, we found the mutants Δ*fapC*, Δ*rpoN*, and Δ*brfA* were defective in producing the biofilm matrixes, and the *fap* genes were positively regulated by BrfA (Figures 3–5). Thus, BrfA is likely to act in conjunction with RpoN to regulate the transcription of *fapA-E*. The regulation mode of BrfA is different from that of the master regulators CsgD in *E*. *coli* and FleQ in *P*. *aeruginosa*, which regulate the expression of biofilm matrices without RpoN (Baraquet et al., 2012; Mika and Hengge, 2014). These results suggest that the Fap amyloids are the main components of the biofilm matrices, and that RpoN and BrfA are essential regulators for the transcription of *fap* genes in PF07.

In this work, several DGC or PDE genes were significantly upregulated or downregulated in the PF07_5d biofilm compared to the PF07_1d biofilm, such as *D7M10_RS04965*, *D7M10_RS06580*, and *D7M10_RS22095* (Table 2). The DGCs or PDEs coded by these genes may regulate the production of biofilm matrices by changing the internal levels of c-di-GMP. In *P*. *aeruginosa* PA14, there were 40 proteins predicted to produce or degrade c-di-GMP, and eight of them take part in regulating the expression of *pel* genes (Okegbe et al., 2017). As the regulation of the DGC and PDE genes to the biofilm matrix formation is often complicated, we intend to study these genes in depth in the future.

In a study of other biofilm matrix proteins, the presence Flp/Tad pili at the surface of bacterial cells was associated with the formation of extremely tenacious biofilms on a variety of solid surfaces in *Actinobacillus actinomycetemcomitans* (Bhattacharjee et al., 2001). Flagella participate in initial attachment to surfaces, migration along the surfaces, and matrix formation of biofilms in some microorganisms (Mika and Hengge, 2014; Fong and Yildiz, 2015). In addition, polysaccharides constitute a predominant fraction of the biofilm matrix, including Psl or Pel polysaccharides in *P*. *aeruginosa* (Fazli et al., 2014), as well as cellulose and PNAG polysaccharides in *E. coli* (Mika and Hengge, 2014). However, in this work, the genes related to Flp/Tad pili, flagella, and polysaccharides were downregulated, rather than upregulated, in the mature macrocolony biofilm of PF07 (Table 2). Therefore, these genes may not be essential for matrix formation of macrocolony biofilms. However, our results show Δ*rpoN* had more severe defects in forming pellicles and SSA biofilms than Δ*fapC* and Δ*brfA* (Figure 4A). This is likely because RpoN also regulates the synthesis of flagella (Table 2), and flagella may be important to the formation of pellicles and SSA biofilms (Klausen et al., 2003; Yamamoto et al., 2012). Moreover, rhamnolipids are synthesized by rhamnosyltransferases RhlA and RhlB in *P*. aeruginosa (Davey et al., 2003). The synthesis of rhamnolipids is regulated by quorum sensing system RhlRI, based on acylated homoserine lactones (Pearson et al., 1997). Low concentration of rhamnolipids increases the affinity for initial adherence of cells to a surface, but the overproduction of rhamnolipids inhibits the formation of mature biofilms (Nickzad and Déziel, 2014). In this work, the expression of gene cluster *rhlABRI* was significantly downregulated in the mature biofilm, and it was upregulated in the *rpoN* mutant. This agrees with the fact that high levels of rhamnolipids are detrimental to the formation of mature biofilms.

The type II secretion (T2S) system directs the transport of a variety of proteins from Gram-negative bacteria, including virulence factors, extracellular enzymes and biofilm matrix proteins (Johnson et al., 2014; Cianciotto and White, 2017). It was reported that the T2S system delivered biofilm matrix proteins RbmA, RbmC, and Bap1 for biofilm formation in *Vibrio cholera* (Johnson et al., 2014). In the present work, the genes for the T2S system were strongly induced in the mature biofilm. The role of the T2S system in PF07 may be similar to that in *Vibrio cholera*. However, the substrates for the T2S system in PF07 need to be further defined.

In addition to biofilm matrix formation, bacterial metabolism was remodeled in adaptation to biofilm growth. According to our results, the genes related to galactonate degradation were significantly upregulated, whereas the genes related to biosynthesis and secretion of extracellular protease and the genes related to arginine degradation were significantly downregulated during the biofilm formation (Table 2). The metabolic alterations may be associated with limited availability of specific nutrients in the biofilm. However, different bacteria show different biofilm metabolism adaptation. For example, in *P*. *aeruginosa*, the genes related to anthranilate degradation were overexpressed in biofilms (Vital-Lopez et al., 2015). In *B*. *subtilis*, the activity of the tricarboxylic acid cycle increased during early pellicle formation, and a shift from fatty acid biosynthesis to fatty acid degradation was detected (Pisithkul et al., 2019).

## Conclusion

In conclusion, the expression of a large set of genes changed significantly during macrocolony biofilm formation, mainly including the genes related to matrix proteins, polysaccharides, rhamnolipids, secretion systems, biofilm regulation, and metabolism. These genes may be involved in biofilm matrix formation directly or indirectly. In addition, we first identified the functional amyloid Fap as the main component of PF07 biofilm matrixes, and the transcription of *fapA-E* genes may be directly regulated by RpoN in conjunction with BrfA. These genes may serve as potential molecular targets for screening new anti-biofilm agents or for biofilm detection in food environments. For example, by qRT-PCR detection of the target genes, the agents that inhibit the transcription of these genes may be screened out, and the formation of the biofilms may be detected quickly.

## Data Availability Statement

The datasets presented in this study can be found in online repositories. The names of the repository/repositories and accession number(s) can be found in the article/Supplementary Material.

## Author Contributions

MG and ST performed the experiments. JZ, AS, and XL analyzed the data. PD revised the manuscript. XL conceived, designed the experiments, analyzed the data, and wrote the manuscript. All the authors read and approved the final version of the manuscript.

## Conflict of Interest

The authors declare that the research was conducted in the absence of any commercial or financial relationships that could be construed as a potential conflict of interest.

## Publisher’s Note

All claims expressed in this article are solely those of the authors and do not necessarily represent those of their affiliated organizations, or those of the publisher, the editors and the reviewers. Any product that may be evaluated in this article, or claim that may be made by its manufacturer, is not guaranteed or endorsed by the publisher.

## Footnotes

1
https://www.ncbi.nlm.nih.gov/nuccore/NZ_CP032618

2
https://www.ncbi.nlm.nih.gov/sra/

## Abbreviations

ANOVA: analysis of variance
c-di-GMP: cyclic diguanylate monophosphate
CLSM: confocal laser scanning microscopy
DAPI: 4′,6-diamidino-2-phenylindole
DEGs: differentially expressed genes
DGC: diguanylate cyclase
5′-RACE: rapid amplification of 5′-cDNA ends
LB: Luria-Bertani
PDE: phosphodiesterases
PNAG: polysaccharide poly-*N*-acetyl-glucosamine
qRT-PCR: quantitative reverse transcription-PCR
SSA: solid surface-associated
TEM: transmission electron microscopy
T2S: type II secretion.
