# Supplementary material for: Genes Involved in Biofilm Matrix Formation of the Food Spoiler Pseudomonas fluorescens PF07
Source: Front Microbiol. 2022 Jun 6;13:881043. doi: 10.3389/fmicb.2022.881043 (PMC9207406; doi:10.3389/fmicb.2022.881043)
Supplement: Supplementary file 1 [file Table_1.DOCX]

**Supplementary Table S1 Primers for mutant construction and 5’-RACE.**

| **Primers** | **Sequence (5’ - 3’)** |
| --- | --- |
| **For *rpoN* deletion** |  |
| 07*rpoN*-MF1 | GGAATCTAGACCTTGAGTCGTTTCGGTAGGCGACATCAAGC |
| 07*rpoN*-MR1 | GTCACATCAATCGCTTACGCTCGGATTGGGTTTTTGCTCTATGTTGT |
| 07*rpoN*-MF2 | ACAACATAGAGCAAAAACCCAATCCGAGCGTAAGCGATTGATGTGAC |
| 07*rpoN*-MR2 | ACAGCTAGCGACGATATGTCTTAGGCGGCAATGAGGAATA |
| 07*rpoN*-TF | GGAGTTCCACATCAACCATATTCG |
| 07*rpoN*-TR | GCAGGACGAACAGCAGGTCAAC |
| **For *fapC* deletion** |  |
| 07*fapC*-MF1 | GGAATCTAGACCTTGAGTCGCGATGGCAGACAGCAATAAC |
| 07*fapC* -MR1 | GGAGTTGGATTGCTGGTTGCCATGGTGTATCTCCTTGCTTCTAA |
| 07*fapC*-MF2 | TTAGAAGCAAGGAGATACACCATGGCAACCAGCAATCCAACTCC |
| 07*fapC*-MR2 | ACAGCTAGCGACGATATGTCGAGTAGCGTTTGTTGCCCAGTA |
| 07*fapC* -TF | AGATCAGCGGGACGATTGAG |
| 07*fapC*-TR | CCTTGTCGTAGCCTTCACCC |
| **For *brfA* deletion** |  |
| 07*brfA*-MF1 | GGAATCTAGACCTTGAGTCGAGCCTTCGCCATTGAGTTCG |
| 07*brfA*-MR1 | CGGTTCAGCACATCGCACAGCCGCAAACCAGGCAATAACT |
| 07*brfA*-MF2 | AGTTATTGCCTGGTTTGCGGCTGTGCGATGTGCTGAACCG |
| 07*brfA*-MR2 | ACAGCTAGCGACGATATGTCGCAATGGCGTATGGTCGGTG |
| 07*brfA*-TF | GGCAGAAGAGGTGGTCAAACG |
| 07*brfA*-TR | GCCGTCCAACACCAACCTCTA |
| **For 5’-RACE** |  |
| *fapA*-R1 | GCCATTGGGCAGGATGGATCGAGTGAT |
| *fapA*-R2 | CCCTGCGAAGTCGTTGTCGCTGAGTTC |
