# Supplementary material for: Genes Involved in Biofilm Matrix Formation of the Food Spoiler Pseudomonas fluorescens PF07
Source: Front Microbiol. 2022 Jun 6;13:881043. doi: 10.3389/fmicb.2022.881043 (PMC9207406; doi:10.3389/fmicb.2022.881043)
Supplement: Supplementary file 2 [file Table_2.DOCX]

**Supplementary Table S2 List of primers used for qRT-PCR.**

| Gene | Gene product description | Primer Sequences (5'to3') | Size of PCR products (bp) |
| --- | --- | --- | --- |
| D7M10_RS00150 | 2-dehydro-3-deoxygalactonokinase | F: GCATTTGCCCTGCGAACCA | 85 |
|  |  | R: GTCGCCACAGACGTCATCAA |  |
| D7M10_RS00760 | poly-beta-1,6 N-acetyl-D-glucosamine export porin PgaA | F: GCCCTATGATTCGCTGTTCGA | 89 |
|  |  | R: GCAGGGCGATCAGGTATTC |  |
| D7M10_RS03060 | Flp family type IVb pilin | F: CGTTCAACTCCAACTGTTC | 70 |
|  |  | R: CGCGACAAGAATCACGTACTCAA |  |
| D7M10_RS03315 | type I secretion system permease/ATPase | F: CTTCAACGCCTCGACCTTGA | 92 |
|  |  | R: CATCGCCCAGGGTTTCCA |  |
| D7M10_RS04965 | GGDEF domain-containing protein | F: GGAACTGGTCAGCGATGGA | 95 |
|  |  | R: GGTGATCGTAGCCCAGCAT |  |
| D7M10_RS06580 | bifunctional diguanylate cyclase/phosphodiesterase | F: CGGTGATGAATTCTGCATCCTGAT | 100 |
|  |  | R: CAGCTCGATGGGCTCTTTCAT |  |
| D7M10_RS08135 | flagellar assembly protein FliT | F: GGGATGCGATCAGCGAATTG | 111 |
|  |  | R: CTAACAGGCTCTCCAGCTTCT |  |
| D7M10_RS10920 | type II secretion system minor pseudopilin GspK | F: GCAAGTTCAACCTGCGCAATC | 132 |
|  |  | R: CAAGCACCCGTTGGCTGAT |  |
| D7M10_RS11565 | GNAT family N-acetyltransferase RhlI | F: CGCCATCACCATGAACTGTCT | 80 |
|  |  | R: CCCAGTTGCTCGATGAACAC |  |
| D7M10_RS13750 | serralysin family metalloprotease | F: GCAGGATCGACTTGACCTACA | 95 |
|  |  | R: CAGGTTGCTGAACTGGCTGAA |  |
| D7M10_RS14105 | glycogen debranching protein GlgX | F: GCCTGATGCCTGACGACAA | 100 |
|  |  | R: GAGTCGGTGACCATTTGCAGTA |  |
| D7M10_RS14465 | FapA | F: CAGATTCTACGCTCGACGACTAA | 83 |
|  |  | R: GATCGAGTGATGCTGGAACCA |  |
| D7M10_RS14470 | FapC | F: CCGTCACCAGCCAGGAATC | 101 |
|  |  | R: GGCATCGTTGTTGACGTAGGTCTT |  |
| D7M10_RS14490 | FapF | F: CCTTGAACGAGCGTATGAGTATG | 109 |
|  |  | R: GCGTCACTGGACACAATCGA |  |
| D7M10_RS14495 | sigma-54-dependent Fis family transcriptional regulator | F: CCTGCCGATGGAGTTGCAA | 105 |
|  |  | R: GCCAACACCCTGACATCCA |  |
| D7M10_RS18830 | hypothetical protein | F: GAGCATCGGACGGAGCTTT | 84 |
|  |  | R: GTCGCTTATCCCCTGGCTAT |  |
| D7M10_RS20365 | flagellar hook protein FlgE | F: CAAGGTTGACTCCAGCAGCAA | 133 |
|  |  | R: GTTGCCTTGAGTGTCATACAAGGT |  |
| D7M10_RS21100 | carbamate kinase | F: GAACAGGAACTGGGCAACCT | 82 |
|  |  | R: GGTCGTTGGCGTCCACTT |  |
| D7M10_RS22095 | GGDEF and EAL domain-containing protein | F: GCGATCAGTTGCTGATCCTGAA | 100 |
|  |  | R: AGCGCATCCTGTGCCAACT |  |
| 16S | internal control | F: GGTGCCTTCGGGAACATTGAGAC | 137 |
|  |  | R: GTCTCCTTAGAGTGCCCACCATTAC |  |
