# Supplementary material for: Genes Involved in Biofilm Matrix Formation of the Food Spoiler Pseudomonas fluorescens PF07
Source: Front Microbiol. 2022 Jun 6;13:881043. doi: 10.3389/fmicb.2022.881043 (PMC9207406; doi:10.3389/fmicb.2022.881043)
Supplement: Supplementary file 7 [file Data_Sheet_1.DOCX]

**
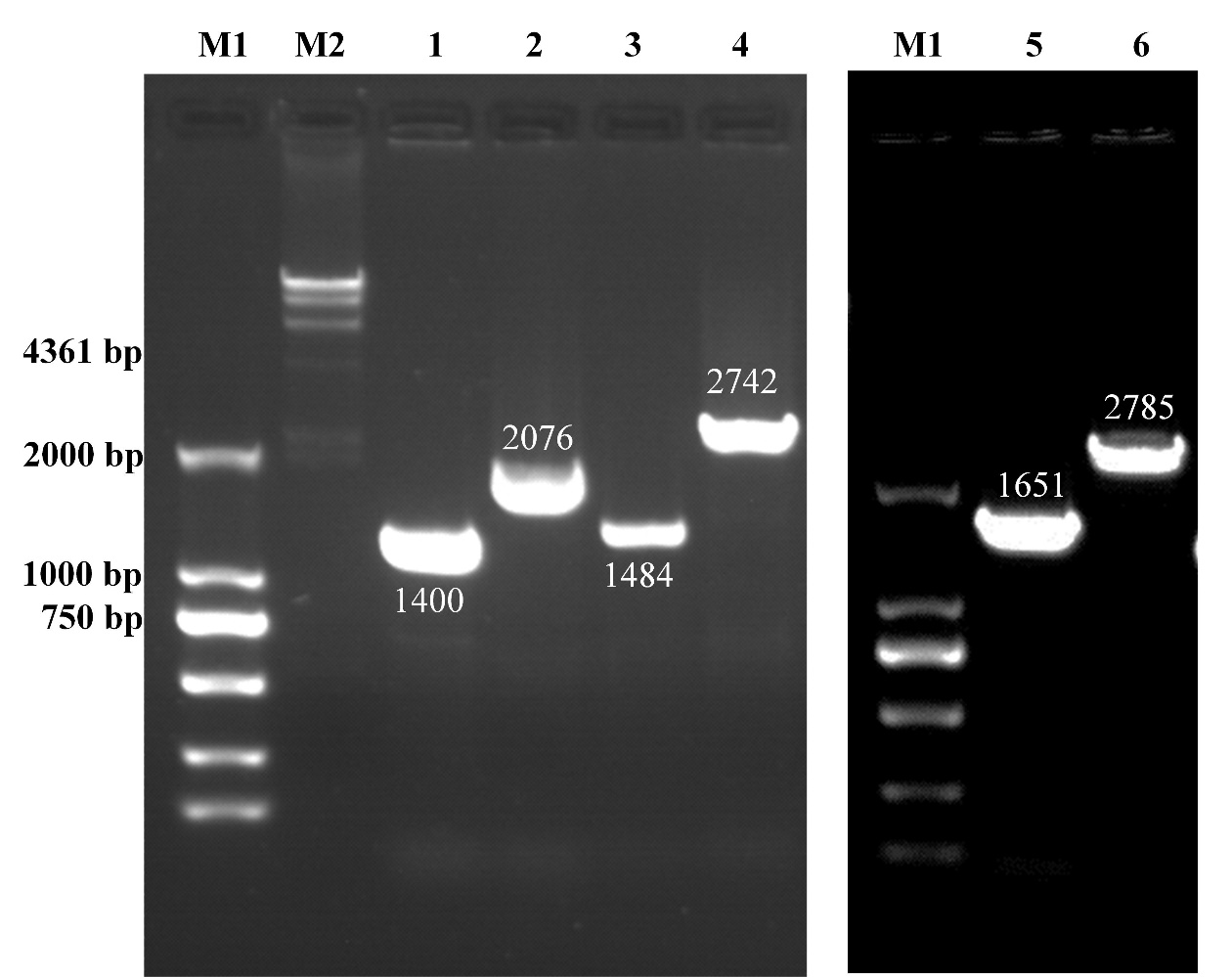
**

**Supplementary Figure S1** PCR determination of *fapC*, *rpoN*, and *brfA* deletion mutants of *P*. *fluorescens* PF07. Lane M1, DL2000 DNA Ladder (Takara, China); Lane M2, λ DNA/Hind III DNA Marker (Takara, China); Lane 1-2, PCR productions of *fapC* mutant and PF07 using external primer pair *fapC*-TF/TR; Lane 3-4, PCR productions of *rpoN* mutant and PF07 using external primer pair *rpoN*-TF/TR; Lane 5-6, PCR productions of *brfA* mutant and PF07 using external primer pair *brfA*-TF/TR.
